# Factors influencing type 2 diabetes in adults: a cross-sectional study

**DOI:** 10.3389/fpubh.2025.1662519

**Published:** 2025-10-07

**Authors:** Yan Zou, Li-chun Huang, Meng-jie He, Dan Han, Dan-ting Su, Pei-wei Xu, Rong-hua Zhang

**Affiliations:** Zhejiang Provincial Center for Disease Control and Prevention, Hangzhou, China

**Keywords:** diabetes mellitus, nutritional status, adults, China, obesity

## Abstract

**Objectives:**

The aim of this study was to explore the factors influencing type 2 diabetes mellitus (T2DM) among adults in Zhejiang Province.

**Methods:**

A stratified cluster sampling technique was employed, and adults without known diabetes were included in the analysis. Food consumption was assessed using three consecutive days of 24-h dietary recall. Blood samples were collected to measure fasting blood glucose (FBG), blood lipids [total cholesterol (TC), triglyceride (TG), low-density lipoprotein cholesterol (LDL-C), high-density lipoprotein cholesterol (HDL-C), 25-(OH)D vitamin D (VD), and vitamin A (VA). Systolic blood pressure (SBP) and diastolic blood pressure (DBP) were also measured. Ordinal regression was used to explore factors influencing T2DM.

**Results:**

The analysis included a total of 5,804 adults. The prevalence rates of T2DM and prediabetes were 5.5 and 5.8%, respectively. Significant differences were observed in age, sex, nutritional status, hypertension, and blood lipid levels among adults with normal fasting blood glucose, prediabetes, and T2DM (*p* < 0.05). Adults aged 55 years and above, those who were overweight or obese, those with hypertension, and those with higher TG levels had a greater risk of developing diabetes (*p* < 0.05).

**Conclusion:**

This study revealed that adults aged 55 years and above, those who are overweight or obese, those with hypertension, and those with higher TG levels have a greater risk of developing diabetes. These findings underscore the need for targeted interventions to manage these risk factors in the prevention and management of T2DM.

## Introduction

Type 2 diabetes mellitus (T2DM) is a major global health priority and is the eighth leading cause of death and disability (1). The 2017 Global Burden of Diseases studies further estimated that high fasting plasma glucose (FPG) was the third most common global risk factor for disability-adjusted life years (2). T2DM is the most prevalent type of diabetes, accounting for more than 90% of diabetes mellitus (DM) cases (3). Diabetes can cause blindness, kidney failure, heart attack, stroke and lower limb amputation. Research has also shown that prediabetes increases the risk of developing T2DM (4). Moreover, prediabetes is associated with an increased risk of cardiovascular disease and all-cause mortality in adults (5). Diabetes has a substantial effect on an individual’s quality of life and places a substantial economic burden on national economies (6). Despite significant progress in the treatment of T2DM, prevention strategies remain crucial given the increasing prevalence of this disease, driven by factors such as population ageing, sedentary lifestyles, and unhealthy dietary habits (7). T2DM is often preventable. Factors that contribute to the development of T2DM include being overweight, insufficient physical activity, and a genetic predisposition.

Given the significant role of nutrition in influencing fasting blood glucose (FBG) levels, prior studies have delved into how various dietary components and patterns impact FBG in adults (8). Some research has indicated that a diet high in refined carbohydrates and saturated fats can lead to elevated FBG levels (9). Conversely, diets rich in fiber, whole grains, and healthy fats have been associated with better glycemic control (10). However, existing findings are inconsistent to some extent and often fail to comprehensively consider the interplay of multiple nutrition factors. Previous Chinese nutrition surveys have provided valuable insights into the general dietary habits and health status of the population but have not fully explored the detailed relationships between specific nutritional factors and T2DM risk in a community-based setting. Our study aims to fill this gap by conducting a more thorough and nuanced analysis of the relationships between nutrition and adult FBG levels. We hypothesize that specific sociodemographic, anthropometric, biochemical, and lifestyle-related factors, particularly nutritional factors, play significant roles in the development of T2DM. This study seeks to explore these factors in depth. The findings of this study can be applied in future public health interventions to reduce the burden and disabilities associated with diabetes.

## Methods

### Study and participants

This study included adults participating in provincial nutrition surveillance, excluding those with a history of T2DM, gestational diabetes, or other specific types of diabetes. A stratified cluster sampling technique was employed. The sampling design was constructed based on the principles of representativeness across regions, sex, and age. In addition to ensuring scientific rigor, the sampling plan also accounted for the feasibility of economic resources and onsite investigations. Streets/townships were designated as the primary sampling units. Stratified sampling was subsequently conducted to recruit participants from different sex and age groups in accordance with their demographic characteristics. The selection of streets/townships was based on census data and geographic information. Within each selected street/township, households were randomly chosen using a systematic sampling method to ensure equal representation of different demographic groups.

All members of the selected households were subsequently interviewed. The field investigation, which included the collection of demographic information, dietary data, and blood samples, was conducted in 2022.

The present study utilized a structured questionnaire from the Chinese Resident Nutrition and Health Survey (CNHS) (11), which collected detailed information on sociodemographic characteristics, daily dietary intake, and daily life behaviours relevant to this study.

### Anthropometric and laboratory assessment

Measurements were conducted by trained investigators using standardized methods in a centralized setting. Height was measured without shoes to the nearest 0.2 cm using a portable stadiometer (TZG, Wuxi weighing instrument factory Co., Ltd., Wuxi, China), and weight was measured without shoes and in light clothing to the nearest 0.1 kg on a calibrated beam scale (G&G tc——200 k, Shanghai Taizhiheng Electronic Weighing Instrument Co., Ltd., Shanghai, China). Body Mass Index (BMI) is the most widely used measure of obesity, and the World Health Organization cut-off is 18.5–23.9 kg/m^2^ for a normal BMI. BMI was calculated as weight (kg)/height (m)^2^. A BMI greater than or equal to 24.0 kg/m^2^ or less than 28.0 kg/m^2^ is considered overweight, whereas a BMI greater than or equal to 28.0 kg/m^2^ is considered obese (12). To assess SBP and DBP, participants rested for 15 min before the measurement. An upper-arm electronic blood pressure monitor was used to measure blood pressure. The diagnostic criteria for hypertension were systolic blood pressure ≥140 mmHg and/or diastolic blood pressure ≥90 mmHg (13).

### Dietary intake calculation

The dietary assessment included three consecutive days of 24-h dietary recall and a concurrent seasoning weighing survey. Family members reported their dietary intake over the prior 24 h, including foods consumed both at home and outside, over three consecutive days, including two weekdays and one weekend day, to estimate daily food and nutrient intake. The seasoning weighing survey assessed household consumption of edible oils, salt, monosodium glutamate, and other major seasonings using the weighing method during the same period, with diner numbers recorded. Both surveys were conducted simultaneously using the Chinese Resident Nutrition and Health Survey (CNHS) questionnaire to ensure accuracy. Daily nutrient intake was calculated using the China Food Composition Table (14).

### Laboratory assessment

Blood samples were collected to measure the concentrations of FBG, blood lipids [including total cholesterol (TC), triglycerides (TG), low-density lipoprotein cholesterol (LDL-C), and high-density lipoprotein cholesterol (HDL-C), vitamin D, and vitamin A]. FBG levels were measured using the hexokinase method. In accordance with the Chinese guidelines for T2DM, FBG ≥ 7.0 mmol/L was used to define diabetes, whereas FBG ≥ 6.1 mmol/L and < 7.0 mmol/L were used to define impaired fasting glucose (IFG) (15). Serum TG levels were measured via a Roche Cobas 8,000 analyser, whereas TC levels were measured via a Hitachi LST008AS analyser. HDL-C and LDL-C levels were measured using the direct method via the Hitachi LST008AS. Abnormal lipid profiles were defined according to the Li et al. (16) as follows:


TG≥1.5mmol/L

TC≥5.2mmol/L

LDL−C≥3.4mmol/L

HDL−C≤1.0mmol/L

Dyslipidaemia was defined as the presence of any one of the following four factors: elevated LDL-C levels, low HDL-C levels, elevated TG levels, or elevated TC levels. Critical lipid profiles were defined according to the Li et al. (16) as follows:


TG≥1.0mmol/L

TC≥4.4mmol/L

LDL−C≥2.8mmol/L

HDL−C≤1.2mmol/L

Vitamin D levels were measured via high-performance liquid chromatography–tandem mass spectrometry (HPLC-MS/MS) (AB SCIEX 5500MPX) as recommended by the Chinese national standard (WS/T677-2020) (17). Vitamin D deficiency and insufficiency were assessed on the basis of the concentration of serum 25-hydroxyvitamin D [25-(OH)D] (WS/T677-2020). Specifically, vitamin D deficiency was defined as a serum 25-(OH)D level less than 12 μg/L. Vitamin D insufficiency was defined as a serum 25-(OH)D level less than 20 μg/L. Vitamin A levels were measured via high-performance liquid chromatography (HPLC) (WS/T553-2017) (18). Vitamin A deficiency and marginal deficiency were assessed on the basis of the concentration of serum retinol, according to the Chinese national standard (WS/T553-2017). Specifically, vitamin A deficiency was defined as a serum retinol concentration less than 0.2 μg/mL. Marginal vitamin A deficiency was defined as serum retinol levels less than 0.3 μg/mL but greater than or equal to 0.2 μg/mL.

### Ethical considerations

All procedures performed in this study were in accordance with the ethical standards of the institutional research committee. Ethical approval was obtained from the Ethics Committee of the Zhejiang Provincial Center for Disease Control and Prevention (approval number: 2022–018-01), and informed consent was obtained from all individual participants included in the study.

### Statistical analysis

Data processing and statistical analyses were performed using SAS 9.2 software. Missing data were handled using multiple imputation techniques. The distributions of DM (FBG ≥ 7.0 mmol/L), IFG (FBG ≥ 6.1 mmol/L and < 7.0 mmol/L), and normal FBG (FBG < 6.1 mmol/L) were analyzed using the chi-square (χ^2^) test. Continuous variables with normal distributions are presented as the means ± standard deviations (SDs). Differences in daily food and dietary nutrient intake among adults with or without DM and IFG were assessed using analysis of variance (ANOVA). Influencing factors were evaluated using ordinal regression models. The outcome variable was IFG status,” which was categorized into three levels: normal fasting glucose (FBG < 6.1 mmol/L), impaired fasting glucose (FBG ≥ 6.1 and <7.0 mmol/L), and DM (FBG ≥ 7.0 mmol/L). On the basis of previous research findings, we identified a set of variables that could confound the relationship between the exposure variables and the outcome. To control for these confounders in the analysis, we first included all the identified potential confounding variables in the univariate analysis. Then, variables with *p* values less than 0.1 in the univariate analysis were included in the ordinal regression model. The initial set of potential confounding variables included age, sex, nutritional status, smoking status, hypertension, and blood lipid levels (TG, TC, HDL-C, LDL-C), vitamin D, and vitamin A. Variables with *p*-values less than 0.1 in the univariate analysis were then included in the ordinal regression model to assess their independent association with IFG status. To address potential seasonal effects and day-to-day variability in the 3-day dietary recall method, data were collected throughout the year, and the recall days included both weekdays and weekends. This approach helps to capture the variability in dietary intake across different seasons and weekdays, ensuring a more comprehensive and representative assessment of dietary patterns. All tests were two-sided, and the level of significance was set at *p* < 0.05.

## Results

### Study participants

A total of 5,804 adults were included in the analysis. Among the 5,940 individuals initially recruited, 136 were excluded due to not meeting the inclusion criteria or having incomplete data for key variables. The final analysis included 5,804 participants with complete data for all variables assessed in the study. The participants had a mean age of 51.4 years (SD, 15.9), with women accounting for 57% of the sample. The characteristics of the participants are presented in Table 1 according to their FBG levels. Of these, 5,149 participants (88.7%) had normal fasting blood glucose (FBG < 6.1 mmol/L), 336 participants (5.8%) had IFG (FBG ≥ 6.1 and <7.0 mmol/L), and 319 participants (5.5%) had diabetes mellitus (FBG ≥ 7.0 mmol/L). Overall, 9.3% of obese adults and 8.2% of adults aged ≥55 years had an FBG ≥ 7.0 mmol/L. As shown in Table 1, significant differences were observed among adults with DM, IFG, and normal FBG when stratified age by (<55 years, ≥55 years) (χ^2^ = 105.483, *p* < 0.001), sex (male, female) (χ^2^ = 14.670, *p* = 0.001), nutritional status (normal weight, overweight, obesity) (χ^2^ = 78.720, *p* < 0.001), smoking status (smokes every day, does not smoke every day, used to smoke, never smoked) (χ^2^ = 14.982, *p* = 0.020), hypertension status (yes, no) (χ^2^ = 48.215, *p* < 0.001), TG level (<1.0 mmol/L, 1.0–1.5 mmol/L, ≥1.5 mmol/L) (χ^2^ = 115.547, *p* < 0.001), TC level (<4.4 mmol/L, 4.4–5.2 mmol/L, ≥5.2 mmol/L) (χ^2^ = 34.489, *p* < 0.001), HDL-C level (≥1.2 mmol/L, 1.0–1.2 mmol/L, <1.0 mmol/L) (χ^2^ = 44.760, *p* < 0.001), LDL-C level (<2.8 mmol/L, 2.8–3.4 mmol/L, ≥3.4 mmol/L) (χ^2^ = 19.940, *p* = 0.001), vitamin D level (≥20 ng/mL, 12–20 ng/mL, <12 ng/mL) (χ^2^ = 11.061, *p* = 0.026), vitamin level A (<0.2 μg/mL, 0.2–0.3 μg/mL, ≥0.3 μg/mL) (χ^2^ = 9.642, *p* = 0.047), and sitting time (<4 h, ≥4 h) (χ^2^ = 6.197, *p* = 0.045).

**Table 1:** Participant characteristics according to fasting blood glucose (*N* = 5,804).

Factors	FPG				
	FBG ≥ 7 mmol/L	6.1 ≤ FBG < 7 mmol/L	FBG < 6.1 mmol/L	*χ* ^2^	*P*
Sex (%)					
Male (*N* = 2,495, 43%)	167 (6.8%)	152 (6.2%)	2,176 (87.0%)	14.670	0.001
Female (*N* = 3,309, 57%)	152 (4.6%)	184 (5.6%)	2,973 (89.8%)		
Nutritional status (%)					
Normal weight	133 (3.9%)	149 (4.4%)	3,086 (91.6%)	78.720	<0.001
Overweight	134 (7.3%)	135 (7.4%)	1,565 (85.3%)		
Obese	52 (9.3%)	52 (9.3%)	458 (81.5%)		
Smoking (%)					
Smoking everyday	58 (7.0%)	48 (5.8%)	717 (87.1%)	14.982	0.020
Smoking but not every day	7 (6.9%)	8 (7.8%)	87 (85.3%)		
Used to smoke	28 (8.5%)	24 (7.3%)	276 (84.1%)		
Never smoked	225 (5.0%)	256 (5.7%)	4,028 (89.3%)		
Age (year)					
<55 (*N* = 3,233, 55.7%)	108 (3.3%)	133 (4.1%)	2,992 (92.5%)	105.483	<0.001
≥55 (*N* = 2,571, 44.3%)	211 (8.2%)	203 (7.9%)	2,157 (83.9%)		
TG (mmol/L)					
<1.0	39 (2.4%)	54 (3.3%)	1,543 (94.3%)	115.547	<0.001
1.0–1.5	48 (3.6%)	77 (5.8%)	1,195 (90.5%)		
≥1.5	167 (8.2%)	167 (8.2%)	1710 (83.7%)		
TC (mmol/L)					
<4.4	72 (3.8%)	97 (5.1%)	1732 (91.1%)	34.489	<0.001
4.4–5.2	102 (5.3%)	104 (5.4%)	1714 (89.3%)		
≥5.2	145 (7.5%)	135 (7.0%)	1,651 (85.5%)		
HDL-C (mmol/L)					
≥1.2	70 (7.8%)	79 (8.9%)	743 (83.3%)	44.760	<0.001
1.0–1.2	79 (6.2%)	90 (7.1%)	1,101 (86.7%)		
<1.0	170 (4.7%)	167 (4.7%)	3,253 (90.6%)		
LDL-C (mmol/L)					
<2.8	153 (5.0%)	159 (5.2%)	2,765 (89.9%)	19.940	0.001
2.8–3.4	88 (5.9%)	80 (5.4%)	1,314 (88.7%)		
≥3.4	78 (6.5%)	97 (8.1%)	1,018 (85.3%)		
VD (ng/ml)					
≥20	8 (2.8%)	12 (4.2%)	269 (93.1%)	11.061	0.026
≥12–20	63 (4.7%)	72 (5.4%)	1,192 (89.8%)		
<12	242 (6.2%)	236 (6%)	3,452 (87.8%)		
VA (μg/ml)					
<0.20	0	1 (33.3%)	2 (66.7%)	9.642	0.047
≥0.20–0.30	0	3 (4.4%)	65 (95.6%)		
≥0.30	165 (5.0%)	197 (6.0%)	2,932 (89.0%)		
Sitting time (hour)					
<4	177 (6.0%)	190 (6.4%)	2,579 (87.5%)	6.197	0.045
≥4	141 (5.1%)	145 (5.3%)	2,472 (89.6%)		
Hypertension					
Yes	66 (8.5%)	63 (8.2%)	643 (83.3%)	48.215	<0.001
No	167 (4.4%)	160 (4.2%)	3,507 (91.5%)		

HDL, high-density lipoprotein; TC, total cholesterol; TG, triglycerides; LDL, low-density lipoprotein; VD,: vitamin D; VA, vitamin A; FBG, fasting blood glucose.

A total of 319 patients were included in the analysis. Among them, the age ranged from 18 to 95 years. The gender distribution was as follows: 167 males (52.3%) and 152 females (47.6%). In terms of body weight classification, 133 patients (41.7%) were of normal weight, 134 patients (42.0%) were overweight, and 52 patients (16.3%) were obese. Regarding lipid parameters, 167 patients (52.3%) had TG levels ≥1.5 mmol/L. For cholesterol indicators, 145 patients (52.4%) presented TC levels ≥5.2 mmol/L, 78 patients (24.5%) had LDL - C levels ≥3.4 mmol/L, and 170 patients (53.3%) showed HDL - C levels ≤1.0 mmol/L.

### Comparison of daily nutrient and food intake among adults with DM, IFG, and normal FBG

Table 2 presents the daily and nutrient food intake according to FBG levels. Significant differences were observed in the intake of dietary fibre among adults with DM, IFG, and normal FBG (*F* = 3.554, *p* = 0.029). Specifically, adults with an FBG ≥ 7.0 mmol/L had a lower intake of dietary fibre. Additionally, adults with FBG ≥ 7.0 mmol/L had lower intakes of vitamin B1 and vegetables, although these differences were not statistically significant (*F* = 2.835, 2.359; *p* = 0.059, 0.095).

**Table 2:** Daily nutrient and food intake according to fasting blood glucose.

	FBG			*F*	*P*
Dietary intake	FBG < 6.1 mmol/L	6.1 ≤ FBG < 7 mmol/L	FBG ≥ 7 mmol/L		
Protein(g)	64.48 ± 29.89	63.80 ± 27.20	66.09 ± 31.59	0.390	0.677
Fat (g)	71.15 ± 34.49	70.68 ± 35.93	73 ± 37.97	0.329	0.719
Carbohydrate (g)	162.68 ± 83.82	163.91 ± 82.80	167.12 ± 95.16	0.306	0.736
Dietary fibre (g)	7.30 ± 6.80	6.20 ± 4.36	6.90 ± 7.62	3.554	0.029
Zn (mg)	8.67 ± 5.22	8.66 ± 4.33	8.62 ± 4.07	0.011	0.989
Fe (mg)	16.39 ± 10.77	16.65 ± 10.82	16.50 ± 8.99	0.077	0.926
Ca (mg)	417.49 ± 330.40	409.55 ± 435.02	415.82 ± 373.91	0.068	0.935
Vitamin C (mg)	68.55 ± 111.128	64.97 ± 47.47	64.59 ± 52.19	0.268	0.765
Vitamin E (mg)	8.55 ± 5.76	8.71 ± 6.11	8.53 ± 5.56	0.094	0.910
Vitamin B1 (mg)	0.77 ± 0.38	0.79 ± 0.44	0.83 ± 0.44	2.835	0.059
Vitamin B2 (mg)	0.69 ± 0.40	0.72 ± 0.86	0.68 ± 0.49	0.583	0.558
Vitamin A (μg RE)	327.54 ± 428.67	341.58 ± 368.89	324.52 ± 328.51	0.145	0.865
Na (mg)	4863.46 ± 6091.712	5312.69 ± 4906.71	4934.78 ± 2917.13	0.710	0.492
K (mg)	1524.64 ± 765.09	1496.13 ± 872.07	1479.33 ± 731.49	0.501	0.606
Mg (mg)	238.63 ± 112.47	237.30 ± 126.87	241.93 ± 104.38	0.111	0.894
Cereals and starchy vegetables(g)	244.82 ± 126.38	241.11 ± 109.02	248.86 ± 138.32	0.227	0.797
Vegetables(g)	242.74 ± 148.54	225.92 ± 121.32	229.29 ± 133.57	2.359	0.095
Fruits(g)	115.06 ± 130.69	106.64 ± 113.72	97.75 ± 88.32	0.650	0.522
Livestock and poultry meat(g)	117.13 ± 80.13	118.15 ± 80.43	124.03 ± 68.88	0.787	0.455
Fish and shrimp(g)	79.69 ± 61.72	78.56 ± 59.56	82.12 ± 63.40	0.178	0.837
Eggs(g)	41.28 ± 46.65	40.67 ± 27.24	40.34 ± 28.67	0.054	0.947
Milk and dairy products(g)	130.6 ± 89.03	123.27 ± 98.20	118.99 ± 82.42	0.506	0.603
Soy products and nuts(g)	22.27 ± 25.29	24.61 ± 41.00	20.54 ± 22.10	1.058	0.347
Fats and oils(g)	32.89 ± 28.71	33.19 ± 32.34	33.87 ± 34.38	0.121	0.886
Salt(g)	7.81 ± 13.04	8.43 ± 7.98	8.17 ± 6.02	0.353	0.702

FBG, fasting blood glucose.

F (ANOVA) refers to the analysis of variance statistic.

### Ordinal regression analysis

We identified potential covariates that may influence the study outcomes. These include age, sex, nutritional status, smoking status, hypertension, and TG, TC, HDL-C, LDL-C, vitamin D, and vitamin A levels. Age was categorized using 55 years as the cut-off, as it reflects the distinct differences in diabetes-related factors observed in our data between younger and older individuals. We constructed several ordinal regression models with different combinations of covariates. After comparing the model fit and parameter estimates, we finalized a model that best represented the data (Table 3).

**Table 3:** Ordinal regression of factors influencing IFG*.

Variable	Category	Reference category	*β*	Wald χ^2^	OR	*p*
Sex	Male	Female	0.017	0.018	1.02	0.895
Age (years)	<55	≥55	0.773	43.856	2.17	<0.001
Nutritional status	Normal weight	Obese	−0.538	10.661	0.58	0.001
	Overweight	Obese	−0.285	3.000	0.75	0.083
Smoking	Smoking every day	never smoked	0.202	1.626	1.22	0.202
	Smoking but not every day	never smoked	−0.247	0.339	0.78	0.560
	Used to smoke	never smoked	0.275	1.969	1.32	0.161
Hypertension	Yes	No	0.491	18.155	1.63	<0.001
TG (mmol/L)	<1.0	≥1.5	−0.839	30.282	0.43	<0.001
	1.0 ~ 1.5	≥1.5	−0.474	12.664	0.62	<0.001
TC (mmol/L)	<4.4,	≥5.2	−0.15	0.554	0.86	0.457
	4.4 ~ 5.2	≥5.2	−0.177	1.185	0.84	0.276
HDL (mmol/L)	≥1.2	<1.0	0.129	0.764	1.14	0.382
	1.0 ~ 1.2	<1.0	−0.07	0.273	0.93	0.602
LDL (mmol/L)	<2.8	≥3.4	−0.147	0.547	0.86	0.460
	2.8 ~ 3.4	≥3.4	−0.208	1.495	0.81	0.221
VD (ng/ml)	≥20	<12	−0.585	3.48	0.56	0.062
	≥12 ~ 20	<12	0.061	0.248	1.06	0.619
VA (μg/ml)	<0.20	≥0.30	0.384	0.107	1.47	0.744
	0.20 ~ 0.30	≥0.30	−0.513	0.699	0.60	0.403
Sitting time	<4 h	≥4 h	0.179	3.023	1.20	0.082

HDL, high-density lipoprotein; TC, total cholesterol; TG, triglycerides; LDL, low-density lipoprotein; VD, vitamin D; VA, vitamin A; IFG, impaired fasting glucose.

*IFG status was categorized into three levels: normal fasting glucose (FBG <6.1 mmol/L), impaired fasting glucose (FBG ≥6.1 and <7.0 mmol/L), and diabetes mellitus (FBG ≥7.0 mmol/L).

Adults aged 55 years and older had a 2.17 times higher risk of having IFG/T2DM compared to those younger than 55 years (OR = 2.17, Wald χ^2^ = 43.856, *p* < 0.001). Normal weight adults had a 0.58 times lower risk of IFG/T2DM compared to obese individuals (OR = 0.58, Wald χ^2^ = 10.661, *p* = 0.001). Adults with hypertension had a 1.63 times higher risk of IFG/T2DM compared to those without hypertension (OR = 1.63, Wald χ^2^ = 18.155, *p* < 0.001). This indicates that hypertension is a significant risk factor for developing IFG/T2DM. Adults with TG levels below 1.0 mmol/L had a 0.43 times lower risk of IFG/T2DM compared to those with levels of 1.5 mmol/L or higher (OR = 0.43, Wald χ^2^ = 30.282, *p* < 0.001). Adults with TG levels between 1.0 and 1.5 mmol/L had a 0.62 times lower risk of IFG/ T2DM compared to those with levels of 1.5 mmol/L or higher (OR = 0.62, Wald χ^2^ = 12.664, *p* < 0.001).

## Discussion

The results of our study revealed several significant factors influencing the presence of T2DM/IFG among adults in Zhejiang Province. These factors include age, nutritional status, hypertension, and TG levels. Specifically, adults aged 55 years and older, those who are overweight or obese, those with hypertension, and those with higher TG levels have a higher risk of developing IFG/T2DM.

### Age and T2DM/IFG

The prevalence of T2DM and prediabetes increases with age. Our study found that adults aged 55 years and older had a 2.17 times higher risk of developing IFG/T2DM compared to younger adults. This finding underscores the need for targeted interventions in older adults to manage risk factors and prevent the progression to T2DM.

The increased risk of developing IFG/T2DM can be attributed to age-related physiological changes, such as a reduction in muscle mass, an increase in visceral fat, and a decline in *β*-cell function and insulin sensitivity (19–22). As individuals age, they typically experience a reduction in muscle mass and changes in visceral fat distribution, both of which contribute to systemic insulin resistance.

### Nutritional status and T2DM/IFG

Our study also identified nutritional status as a significant risk factor. Specifically, normal weight adults had a 0.6 times lower risk of IFG/T2DM compared to those with obesity, while overweight adults had a 0.8 times lower risk. These findings are supported by previous research indicating that obesity is the most significant modifiable risk factor for developing T2DM (23). For instance, a study by Xiong H highlighted the strong correlation between obesity and diabetes, suggesting that excess body fat contributes to insulin resistance and chronic inflammation, which can exacerbate metabolic dysfunction (24). And obesity is more sensitive than BMI as an early indicator of chronic disease (25).

### Hypertension and T2DM/IFG

Hypertension was another significant risk factor identified in our study. Adults with hypertension had a 1.6 times higher risk of IFG/T2DM compared to those without hypertension. This finding aligns with numerous studies that have established the comorbidity of hypertension and diabetes. For example, a study by Hu X found that a large proportion of individuals with T2DM also develop hypertension (26). The shared pathophysiological mechanisms include insulin resistance and the effects of elevated blood pressure on pancreatic islet function (27). Weight-management-based strategies should be promoted for the prevention of diabetes mellitus. A previous study, which was conducted as part of the China Patient-Centred Evaluative Assessment of Cardiac Events Million Persons Project and included 898,929 young and middle-aged participants, reported a prevalence of hypertension of 26.15% (28). Appropriate attention should be given, and effective policies should be implemented to mitigate the increasing trends of diabetes, prediabetes, and hypertension among adults.

### Dietary factors and T2DM/IFG

The associations between dietary nutrients and blood glucose levels provide valuable insights into the potential dietary factors influencing glycaemic control. The significant difference in dietary fibre intake among adults with DM, IFG, and normal FBG highlights the importance of fibre in blood glucose management. Dietary fibre is known to slow carbohydrate absorption and improve insulin sensitivity (29), which may explain the observed pattern of lower fibre intake in individuals with higher FBG levels. The trend of lower vitamin B1 and vegetable intake in adults with FBG ≥ 7.0 mmol/L, although not statistically significant, aligns with previous research suggesting that these nutrients play a role in glucose metabolism. These findings underscore the need for further investigation into how specific dietary components can be optimized to support better glycaemic outcomes. Consistent with our findings, a study by Danielle et al. (30) demonstrated that higher dietary fibre intake was associated with improved glycaemic control. Tang et al. reported that increased vegetable consumption was linked to a reduced risk of developing T2DM (31). These studies reinforce the importance of dietary factors in diabetes prevention and management.

A study revealed that uncontrolled T2DM is associated with high levels of LDL, TG, and TC, regardless of differences in food habits and lifestyles (32). In this study, elevated TG levels were also found to be associated with an increased risk of IFG/T2DM. Adults with TG levels below 1.0 mmol/L had a 0.4 times lower risk of IFG/T2DM compared to those with TG levels of 1.5 mmol/L or higher, while adults with TG levels between 1.0 and 1.5 mmol/L had a 0.6 times lower risk. This finding is supported by previous research that has highlighted the role of dyslipidemia in the development of insulin resistance and metabolic syndrome (33). Another study revealed a nonlinear association between the TG/HDL-C ratio and the likelihood of diabetes in nonobese individuals from East Asia (34). Dehzad reported elevated TG levels in individuals with diabetes (35). This consistency across studies underscores the significant role of lipid metabolism in diabetes pathogenesis. When comparing the determinants of elevated FBG in non-diabetic adults with established risk factors for T2DM, several biological and behavioral distinctions emerge. Biologically, elevated FBG in non-diabetic adults may reflect early stages of *β*-cell dysfunction and insulin resistance, which are precursors to T2DM. However in non-diabetic individuals, these metabolic alterations may not yet have reached the threshold for a diabetes diagnosis. Behaviorally, non-diabetic adults with elevated FBG may exhibit dietary patterns and lifestyle habits that resemble those of individuals at risk for T2DM, such as high intake of refined carbohydrates and low levels of physical activity. However, the extent and duration of these behavioral risk factors may differ between the two groups.

### Public health implications

The findings of this study have several implications for public health and community settings in Zhejiang Province. Targeted prevention strategies should focus on older adults, those who are overweight or obese, and individuals with hypertension. Specific recommendations include:

Promoting healthy eating habits: Encourage the consumption of high-fiber foods, whole grains, and vegetables.
Encouraging physical activity: Promote regular exercise to manage weight and improve overall health.
Regular health check-ups: Implement routine screening for T2DM/IFG, especially in high-risk groups.
Integrated management: Ensure that primary care settings provide comprehensive management for hypertension and diabetes.

Our study has several strengths. First, this is the first study to examine the factors influencing prediabetes and diabetes through community nutrition surveillance in Zhejiang Province. The sample used in this study was obtained using a stratified cluster sampling technique, which ensures that it is representative of the population in the region. Nonetheless, our study also has several limitations that should be acknowledged. The cross-sectional design limits our ability to establish causality between these factors and IFG/prediabetes. Additionally, we did not perform an oral glucose tolerance test (OGTT), which is considered the gold standard for diagnosing T2DM and IFG. The absence of OGTT results may have led to some misclassification of diabetes and IFG cases, potentially underestimating the true prevalence and associated risk factors. Future research should incorporate OGTTs to improve diagnostic accuracy. We acknowledge that our study’s focus on non-diabetic individuals and single - measurement FBG levels limits causal inference. FBG variability and longitudinal trends were not addressed in this study. Future research should incorporate multiple FBG measurements and longitudinal data to better understand the causal relationships and progression of diabetes. Dietary intake data were obtained through participant recall, which may introduce recall bias and inaccurate reporting of dietary habits. Participants may forget certain foods or misreport portion sizes, leading to discrepancies between reported and actual intake. This recall bias could influence the observed associations between dietary components and glycaemic outcomes. Furthermore, while we identified several influencing factors, there may be other unmeasured variables that also play a role in the development of IFG and prediabetes. However, the characteristics of age, nutritional status, hypertension, and triglyceride levels observed in our study are common factors influencing diabetes and prediabetes globally. Therefore, these findings may be generalizable to other populations beyond Zhejiang Province. However, it should be noted that differences in dietary habits, genetic factors, and the availability of health care resources across different regions may affect the generalizability of these findings. Future studies are encouraged to explore the generalizability of these findings through multicentre studies.

## Conclusion

The results of this study demonstrate that adults aged 55 years and above, those who are overweight or obese, those with hypertension, and those with higher TG levels have a greater risk of IFG.

Given that T2DM is a major global health priority, interest in the prevention of T2DM and prediabetes has increased. As suggested by our study findings, controlling BMI and preventing hypertension and elevated TG levels are vital for the prevention of IFG.

## Data Availability

The ethics committee confirmed that the raw data cannot be shared with third parties, meaning we are unable to provide the raw data directly. Requests for further information should be directed to RZ, yingyang901@yeah.net.
